# Supplementary material for: Antimicrobial Usage and Antimicrobial Resistance in Commensal Escherichia coli from Broiler Farms: A Farm-Level Analysis in West Java, Indonesia
Source: Antibiotics (Basel). 2024 Dec 5;13(12):1181. doi: 10.3390/antibiotics13121181 (PMC11672412; doi:10.3390/antibiotics13121181)
Supplement: Supplementary file 1 [file antibiotics-13-01181-s001.zip › File S1 Overview of AMU per cycle per farm.pdf]

Farm ID	Cycle	Amoxicillin (penicillin (HIA))	Colistin (polymyxin (HPCIA))	Ciprofloxacin (fluoroquinolone (HPCIA))	Doxycycline (tetracycline (HIA))	Enrofloxacin (fluoroquinolone (HPCIA))	Erythromycin (macrolide (HPCIA))	Flumequine (quinolone (HPCIA))	Fosfomycin (phosphonic derivates (HPCIA))	Lincomycin (lincosamide (HIA))	Neomycin (aminoglycoside (CIA))	Oxytetracycline (tetracycline (HIA))	Spectinomycin (aminocyclitol (IA))	Spiramycin (macrolide (HPCIA))	Sulfadiazine (sulfonamide (HIA))	Sulfaquinoxaline (sulfonamide (HIA))	Trimethoprim (trimethoprim (HIA))	Tylosin (macrolide (HPCIA))	Total use ($TF_{count\text{-}based}$)[2]
TOTAL[3]	78	116	112	16	79	183	79	19	16	12	16	51	12	32	44	14	44	74	919
1	1	3	3		3	3	3												0,50
	2	3	3		6		6												0,60
	3	3	3	3	4	6	4											4	0,90
	4	3	3	2	1		1												0,33
2	1	3	3			8													0,47
	2			4		3													0,23
	3					1													0,03
	4	2	2																0,13
3	1					6									5		5	2	0,60
	2					8									3		3	3	0,57
	3					8									3		3	4	0,60
	4					10									3		3	5	0,70
4	1					7									3		3	3	0,53
	2					7									3		3	3	0,53
	3					7									3		3	3	0,53
	4					7									3		3	3	0,53
	5			4		3									3		3	7	0,67

5	1	4	4																0,27
	2	4	4		4		4												0,53
	3									4	4	4	4						0,53
	4				2		2			4	4	4	4						0,67
6	1				4		4												0,27
	2					4													0,13
	3										4	11							0,50
	4										4	4							0,27
7	1					4													0,13
	2			2														2	0,13
	3			1	2		2											1	0,20
	4	3																	0,10
8	1	4	4																0,27
	2	4	4			9													0,57
	3	3	3																0,20
	4	3													7		7		0,57
9	1	4			4		4												0,40
	2				5		5		5									5	0,67
	3				3		3		6									6	0,60
	4				4		4		5									5	0,60
10	1	3	3																0,20
	2	3	3																0,20
	3							4						4					0,27
	4							4						4					0,27
11	1	3	3																0,20
	2							3						3					0,20
	3							4						4					0,27
	4							4						4					0,27

12	1		2											2	5		5		0,47
	2	3	3																0,20
	3	3	3																0,20
	4	4	4																0,27
13	1														3		3		0,20
	2	1	1																0,07
	3	3	3																0,20
	4	5	5																0,33
14	1				4	10	4											4	0,73
	2				4	10	4											4	0,73
	3				4	9	4											3	0,67
	4				4	10	4											4	0,73
15	1	3			3	3	3												0,40
	2	3	3		3	4	3												0,53
	3	3			3	3	3												0,40
	4	4	4		4	3	4												0,63
	5					3						4							0,23
16	1	3	7			3								4					0,57
	2	3	7			3								4					0,57
	3	3				3													0,20
	4		3			3								3					0,30
17	1	3	6		4	5	4											3	0,83
	2	1	1		1	2	1												0,20
	3	1	1		2	3	2												0,30
	4	1			1	2	1												0,17
18	1											2				5			0,23
	2											6				3			0,30
	3	5	5									5							0,50

	4									4		1	4			2			0,37
19	1	3	3									2				4			0,40
	2	3	3									3							0,30
	3	3	3									2							0,27
	4					3						3							0,20

[1] Per antimicrobial the number of treatment days that it was used is shown per cycle per farm. For each antimicrobial that was used, the antimicrobial class and the classification according to the WHO to its importance for human health is added between brackets [16].

[2] Sum of treatment days with antimicrobials per cycle per farm expressed in $TF_{count\text{-}based}$.

[3] Sum of treatment days per antimicrobial across 78 production cycles.
