# Supplementary material for: Antimicrobial Usage and Antimicrobial Resistance in Commensal Escherichia coli from Broiler Farms: A Farm-Level Analysis in West Java, Indonesia
Source: Antibiotics (Basel). 2024 Dec 5;13(12):1181. doi: 10.3390/antibiotics13121181 (PMC11672412; doi:10.3390/antibiotics13121181)
Supplement: Supplementary file 1 [file antibiotics-13-01181-s001.zip › File S3 A description of the EUVSEC plate that was used for antimicrobial susceptibility testing.pdf]

1311201135

SENSITITRE

EUVSEC

Veterinary Reference Card

	1	2	3	4	5	6	7	8	9	10	11	12
A	SMX 1024	TMP 32	CIP 8	TET 64	MERO 16	AZI 64	NAL 128	CHL 128	TGC 8	COL 16	AMP 64	GEN 32
B	SMX 512	TMP 16	CIP 4	TET 32	MERO 8	AZI 32	NAL 64	CHL 64	TGC 4	COL 8	AMP 32	GEN 16
C	SMX 256	TMP 8	CIP 2	TET 16	MERO 4	AZI 16	NAL 32	CHL 32	TGC 2	COL 4	AMP 16	GEN 8
D	SMX 128	TMP 4	CIP 1	TET 8	MERO 2	AZI 8	NAL 16	CHL 16	TGC 1	COL 2	AMP 8	GEN 4
E	SMX 64	TMP 2	CIP 0.5	TET 4	MERO 1	AZI 4	NAL 8	CHL 8	TGC 0.5	COL 1	AMP 4	GEN 2
F	SMX 32	TMP 1	CIP 0.25	TET 2	MERO 0.5	AZI 2	NAL 4	FOT 1	TGC 0.25	TAZ 2	AMP 2	GEN 1
G	SMX 16	TMP 0.5	CIP 0.12	CIP 0.03	MERO 0.25	MERO 0.06	FOT 4	FOT 0.5	TAZ 8	TAZ 1	AMP 1	GEN 0.5
H	SMX 8	TMP 0.25	CIP 0.06	CIP 0.015	MERO 0.12	MERO 0.03	FOT 2	FOT 0.25	TAZ 4	TAZ 0.5	POS CON	POS CON

ANTIMICROBICS

SMX	Sulfamethoxazole
TMP	Trimethoprim
CIP	Ciprofloxacin
TET	Tetracycline
MERO	Meropenem
AZI	Azithromycin
NAL	Nalidixic Acid
FOT	Cefotaxime
CHL	Chloramphenicol
TGC	Tigecycline
TAZ	Ceftazidime
COL	Colistin
AMP	Ampicillin
POS	Positive Control
GEN	Gentamicin

# Indicates Sensititre® range where different to CLSI M100/M31/M45. For current Quality Control ranges refer to QC range document.

Sensititre® Bereich wo unterschiedlich zu CLSI M100/M31/M45. Für gegenwärtige Qualitätskontrolle beziehen sich Strecken auf QC Strecke Dokument.

Indica el rango esperado con Sensititre® en caso de diferir del establecido por el CLSI M100/M31/M45. Para el control de calidad actual las gamas refieren al documento de la gama de QC.

Indicare il range del Sensititre® laddove e differente dal CLSI M100/M31/M45. Per controllo di qualità corrente le gamme si riferiscono al documento della gamma di QC.

Δείχνει τη σειρά Sensititre® όπου διαφορετικός σε CLSI M100/M31/M45. Για τον τρέχοντα ποιοτικό έλεγχο οι σειρές αναφέρονται qc στο έγγραφο σειράς.

Indique où la gamme Sensititre® est différente à CLSI M100/M31/M45. Pour les gammes actuelles du contrôle Qualité, se referer au document de gamme de QC.

File S3: A description of the EUVSEC plate that was used for antimicrobial susceptibility testing
